# EZH2 dependent epigenetic landscape in adult T cell leukemia and Tax immortalized cells

**DOI:** 10.1186/1742-4690-12-S1-O13

**Published:** 2015-08-28

**Authors:** Dai Fujikawa, Makoto Yamagishi, Shota Nakagawa, Naoya Kurokawa, Ai Soejima, Seiichirou Kobayashi, Kaoru Uchimaru, Yuetsu Tanaka, Kazumi Nakano, Toshiki Watanabe

**Affiliations:** 1 Graduate School of Frontier Sciences, The University of Tokyo, Tokyo, Japan; 2 Institute of Medical Science, The University of Tokyo, Tokyo, Japan; 3 Graduate School and Faculty of Medicine, University of the Ryukyus, Okinawa, Japan

## 

Epigenetic regulations globally determine gene transcription. Recent studies have revealed that expression changes and genetic mutations of epigenetic factors cause epigenetic imbalance in cancers. We previously reported that aberrant expression of Polycomb repressive complex 2 (PRC2) components causes constitutive NF-κB activation through silencing of miR-31 in ATL cells. However, the underlying mechanisms by which the epigenetic imbalance is induced and maintained remain to be elucidated. Here, we conducted ChIP-on-chip and transcriptome analyses of ATL and normal CD4+ T cells and found that the epigenetic reprogramming closely associates with ATL specific gene expression signature. Leukemic cell-specific silencing of cell cycle regulator, CDNK1A, was correlated with H3K27me3 level. In addition, orchestrated loss of microRNAs and multiple transcription factors appears to be mediated by the epigenetic mechanism. EZH2 upregulation and H3K27me3 accumulation were found in HTLV-1-infected populations derived from ATL patients as well as asymptomatic carriers. Furthermore, EZH2 inhibition blocked Tax-dependent cell growth and its immortalization in vitro. Intriguingly, the Tax-triggered immortalizing cells partially mimicked the methylation pattern observed in ATL cells, suggesting that the epigenetic alterations are closely involved in immortalization of infected cells and disease progression. In parallel, we found that over expression of PRC2 core components was induced by active signaling cascades including NF-κB in ATL cells. Collectively, our results suggest a coherent positive feedback mechanism comprised of PRC2 and NF-κB signaling, which stabilizes the epigenetic rearrangement and phenotypic outcomes in HTLV-1 infected cells. Since pharmacological inhibition of EZH2 selectively killed ATL and HTLV-1 infected cells in ex vivo culture, targeting the epigenetic elements will hold great promise in treatment and prevention of ATL and HTLV-1-related diseases.

